# Tolerability of high intensity interval training (HIIT) in individuals living with knee OA and at risk for cardiovascular disease: A prospective cohort study (THIPO)

**DOI:** 10.1016/j.ocarto.2025.100712

**Published:** 2026-01-12

**Authors:** Mathilde Espe Pedersen, Thomas Bandholm, Mathias Ried-larsen, Cecilie Bartholdy, Tanja Schjødt Jørgensen, Asbjørn Seenithamby Poulsen, Kasper Stagberg Madsen, Marius Henriksen

**Affiliations:** a The Parker Institute, Copenhagen Univ. Hosp. Bispebjerg Frederiksberg, Copenhagen, Denmark; b Physical Medicine & Rehabilitation Research - Copenhagen (PMR-C), Department of Department of Physical and Occupational Therapy, Copenhagen Univ. Hosp., Amager Hvidovre, Denmark; c Dept. of Clinical Research, Copenhagen Univ. Hosp. Amager Hvidovre, Denmark; d Dept of Orthopedic Surgery, Copenhagen Univ. Hosp. Amager Hvidovre, Denmark; e Department of Clinical Medicine, University of Copenhagen, Copenhagen, Denmark; f Centre for Physical Activity and Research (CFAS), University Hospital Rigshospitalet, Novo Nordisk A/S, Denmark

**Keywords:** Knee osteoarthritis, Exercise, Aerobic exercise

## Abstract

**Objective:**

To explore the tolerability of a 12-week High Intensity Interval Training (HIIT) program in individuals with knee osteoarthritis (OA) at elevated risk for cardiovascular disease (CVD).

**Methods:**

Forty-one individuals with knee OA and elevated CVD risk participated in a 12-week HIIT program with three weekly supervised sessions (36 in total) on treadmills, cross-trainers, row- and cycle ergometers. The program comprised a 10-min warm-up, followed by eight 2-min intervals of high intensity (>80 % of HRmax) followed by 2 min of moderate training intensity (60 % of HRmax), and 5 min of cool down. Tolerability was assessed by investigator consensus using a decision guiding framework based on attendance, incidence of knee pain flares resulting in non-compliance with the HIIT program during the 12-week period and change in knee pain (0–10 scale) at session start from the first to the last week of the program.

**Results:**

The average Borg Rating of Perceived Exertion (RPE) score during intervals was 16.2 (SD 1.2) over the 12 weeks. Average attendance to sessions was 88 %. The prevalence of knee flares increased from 3 to 5 from the first to the last 6 weeks, and the number of participants with ≥1 point knee pain reduction from week 1 to week 12 was 19 (46 %). According to KOOS thresholds, participants had mild difficulty in Pain, ADL, and Symptoms; moderate difficulty in Knee-related Quality of Life; and moderate-to-severe difficulty in Function in Sports and Recreation.

**Conclusion:**

In conclusion, the HIIT program appeared tolerable for most individuals with knee OA and elevated CVD risk including those with mild-to-moderate difficulties in pain, function, and quality of life, supporting its potential feasibility in this clinical population.

## 1 Introduction

Knee osteoarthritis (OA) is common with joint pain as the primary symptom causing functional limitations, poor sleep, fatigue, depressed mood, and loss of independence [1,2]. Exercise is a first-line treatment for knee OA and should be offered to all patients, regardless of age, comorbidity, or disease severity [[1], [2], [3], [4]]. However, many aerobic exercise interventions for knee OA do not meet the intensity or duration recommended in physical activity guidelines and therefore may not elicit meaningful cardiovascular improvements [3,5,6]. The current exercise paradigm focuses on muscle strengthening of the periarticular muscles (acting directly on the knee joint), and neuromuscular exercises (biomechanical approach aiming to optimize movement patterns) as the predominantly applied exercise modalities [7,8].

Almost 9 out of 10 individuals with knee OA have at least one co-morbidity and are at increased risk of cardiovascular disease (CVD) and premature mortality [9,10], yet current recommendations for knee OA management rarely consider reduction of CVD risks as an explicit target for exercise interventions [3,4,11,12]. Aerobic exercise in the form of High-Intensity Interval Training (HIIT) effectively increases peak oxygen uptake (VO2peak) and can reduce risk of CVD and all-cause mortality in healthy adults [11,[13], [14], [15]]. HIIT is characterized by a number of short bouts of near-maximal to maximal intensity separated by periods of lower intensity [16].

Evidence supports aerobic exercise programs for improving knee OA-related symptoms and physical function [1,3,4,12,17]. However, clinicians often express concerns about HIIT due to a perceived lacking ability of patients to adhere to the high intensity, fear of symptom exacerbations, worsened joint health, and overall safety [[18], [19], [20]]. While concerns about the tolerability of HIIT in patients with knee OA are reasonable, evidence remains limited. One pilot study assessed a 12-week HIIT program and reported modest improvements in VO2peak and high exercise enjoyment [20]. But the tolerability of HIIT at intensities sufficient to induce meaningful cardiorespiratory adaptations remains unverified [20]. The aim of this study was to evaluate the tolerability of 12-week HIIT program in patients with knee OA and at least one CVD risk factor.

## 2 Materials and methods

### 2.1 Study design

In this prospective cohort study, patients with knee OA participated in a supervised 12-week HIIT program at The Parker Institute, Bispebjerg and Frederiksberg Hospital, Copenhagen, Denmark. Testing and assessment were conducted at baseline and week 12. No blinding was applied in the study. The study was approved by the Health Research Ethical Committee of the Capital Region of Denmark (H-22064954) and prospectively registered at clinicaltrials.gov (NCT05777564) on September 2nd, 2023. Reporting of the study follows the Strengthening the Reporting of Observational Studies in Epidemiology guidelines and the Consensus on Exercise Reporting Template (checklist in Supplement 1). All participants provided written informed consent.

### 2.2 Participants

Adults >18 years with a diagnosis of knee OA (American College of Rheumatology (ACR) clinical classification criteria) [21], at least one CVD risk factor (obesity: Body mass index ≥30 kg/m2, hypertension: Systolic ≥135 and/or diastolic ≥85 mmHg, elevated HbA1c: ≥7.25 mmol/L, elevated triglycerides: ≥1.7 mmol/L, or elevated LDL cholesterol: ≥3.0 mmol/L), and knee pain ≥4 (0–10 Numeric Rating Scale, NRS), were considered eligible. Main exclusion criteria were: contraindications to exercise (e.g., resting systolic blood pressure >200 mmHg, diastolic blood pressure >115 mmHg, or acute/recurrent chest pain); a major CVD event within the past five years; current engagement in ≥150 min/week of moderate-intensity or ≥75 min/week of vigorous-intensity physical activity; and inflammatory joint diseases.

#### 2.2.1 High intensity interval training (HIIT)

The HIIT program consisted of a singular education and a 12-week supervised HIIT program.

##### 2.2.1.1 Education session

The education session lasted 45-min and was delivered by a physiotherapist and exercise physiologist. It focused on physiological effects of aerobic exercise, dietary recommendations during an exercise program, potential experience of delayed onset muscle soreness, management of potential knee OA symptom flares, and instructions in how to monitor exercise intensity using Borg Rating of Perceived Exertion (RPE); 6 = rest and 20 = maximal effort) [22] during the HIIT sessions.

##### 2.2.1.2 HIIT exercise program

The HIIT was delivered by a physiotherapist and exercise physiologist in 45-min supervised sessions 3 times/week for 12 weeks (36 in total) in groups of 6–12 participants. The program comprised a 10-min warm-up, followed by eight 2-min intervals of high intensity (aiming at Borg RPE of at least 16 – corresponding to a heart rate of >80 % of HRmax) followed by 2 min of moderate training intensity (aiming at Borg RPE of 11 corresponding to heart rate of at least 60 % of HRmax), and 5 min of cool down. In the first two weeks of the HIIT program a familiarization to the program occurred (i.e., Borg RPE <16 during the intervals was allowed in this period) to ensure high exercise quality and motivation in the remaining part of the exercise program. At each exercise session, all participants rated their perceived exertion using the 6–20 Borg RPE [22] to evaluate exercise intensity after the fourth of the 8 HIIT intervals. If the participant was able to talk during intervals (Borg RPE <16), the participant was encouraged to increase either speed or resistance.

The HIIT was performed on treadmills, cross-trainers, row- and cycle ergometers, as the participants preferred. This approach facilitated individualization, accommodating differences in physical ability, joint discomfort, and aimed to enhance adherence by involving participants in the choice of modality. Each session was recorded as completed (yes/no), together with type of equipment.

##### 2.2.1.3 Modification to the exercise program

If a participant reported knee pain of ≥5 before or during a session, the exercise was moderated to maintain the prescribed exertion level while lowering joint load. In case of a pain flare, modifications were applied in subsequent sessions. If the pain was considered normal by the participant, the session proceeded as planned unless symptoms worsened, prompting adjustments. Modifications to the program included reducing resistance, adjusting speed, or substituting exercise equipment to maintain the target exertion level while minimizing joint discomfort. This approach ensured the intervention remained both standardized and responsive to individual needs.

#### 2.2.2 Tolerability assessment

According to The United States Food and Drug Administration (FDA), ‘tolerability’ refers to the patient's ability to endure an intervention without experiencing unacceptable side effects or adverse reactions [23]. This is vital for improving patient compliance, outcomes and aids in understanding the intervention's feasibility and practical application [24].

In this study we defined tolerability as a composite assessment of three outcomes in a decision guidance framework decided a priori: 1) Attendance to the intervention; 2) incidence of knee pain flares resulting in non-compliance or modification of the HIIT program; and 3) change in knee OA pain.

Adherence was evaluated based on the number of exercise sessions attended (as a proxy for disruptions to daily life). Knee pain flares was evaluated as a symptomatic exacerbation between exercise sessions resulting in non-compliance with or modification of the HIIT program. For each participant the change in knee OA pain was calculated as the difference between the knee pain (0–10 NRS) when starting an exercise session in the first (week 1) and the last week (week 12). At all sessions the participants reported knee pain on a 0–10 NRS before, during (halfway in the HIIT program), immediately after, and 24 h after each exercise session [25]. Participants were asked the following questions: *What is your knee pain on a scale from 0 to 10 (0 is no pain and 10 is the worst imaginable) [before the/during the/after the/24 h after the most recently completed] exercise session?*

The intervention was deemed “tolerable”, “partly tolerable”, or “not tolerable” based on an interpretation made by the investigators guided the decision guiding framework shown in Table 1. The investigators included medical doctors, physiotherapists and exercise physiologists. To enhance the robustness of this assessment, a consensus-based approach was employed. Each investigator independently evaluated each criterion and assigned a classification of “tolerable,” “partly tolerable,” or “not tolerable,” providing detailed justifications through free-text explanations. The documented individual assessments served as All pa foundation for the consensus discussion, ensuring a comprehensive interpretation of the intervention's tolerability.

**Table 1:**
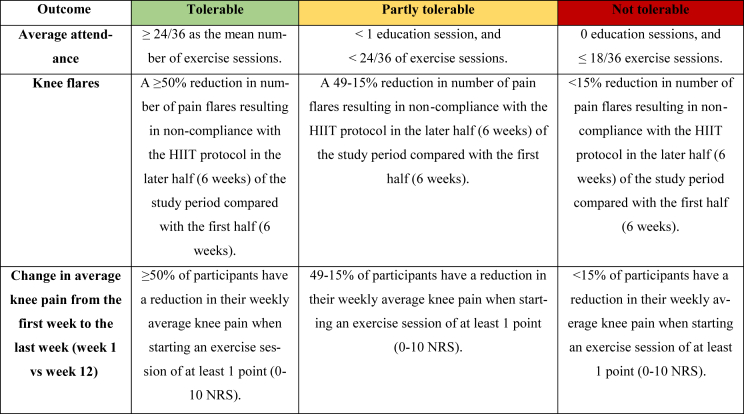
Decision guiding framework.

This table categorizes outcomes from exercise-based interventions for participants with knee osteoarthritis into three levels of tolerability: Tolerable (green), Partly tolerable (yellow), and Not tolerable (red). The table presents criteria for average attendance, knee flares, and change in weekly knee pain during the 12-week intervention.

#### 2.2.3 Knee OA symptoms

At baseline and after the 12 weeks, knee OA symptoms were assessed using the KOOS questionnaire [26]. The KOOS consists of 42 items with subscales of Pain, Function, Knee related quality of life, Symptoms, and Sports and Recreation. A normalized score is calculated for each subscale with 100 indicating no symptoms and functional impairment and 0 indicating extreme symptoms and functional impairment.

#### 2.2.4 Blood pressure and markers of CVD risk factors

Systolic and diastolic blood pressures were measured using a standard blood pressure apparatus and measured at baseline and week 12. A fasting blood sample (10 ml) were analyzed for triglycerides, LDL cholesterol, glucose, and HbA1c at baseline and week 12.

#### 2.2.5 Cardiorespiratory fitness

Cardiorespiratory fitness was assessed by a VO2max test (VO_2_max), at baseline and week 12 by a cardiopulmonary exercise test (43,44) on an ergometer bicycle. A bicycle with breath-by-breath gas analysis ((Vyntus® CPX, Vyaire Medical GmbH, Hoechberg, Germany)) with an individual load protocol with a gradual increase (1 W every second) in workload until exhaustion, was used. Participants performed a 5-min warm-up, to accustom them to the bicycle. An estimated increase in load with expected peak after 10 min was used. The test continued until the following criteria were met: a target VO2max between 8 and 12 min, a respiratory exchange ratio (RER) ≥1.05 [27] and Borg RPE >16 ^25^ Participants completed the test with a 5-min cooldown recovery.

#### 2.2.6 Analgesics

The participants’ use of over-the-counter paracetamol and ibuprofen was recorded during a clinical assessment with an investigator at baseline and week 12.

#### 2.2.7 Safety

In this study, we have adopted the ICH definition of adverse event (AE) and serious adverse event (SAE) [28] AEs/serious adverse events (SAEs) were recorded during the 12-week intervention period. The relationship of the AEs to the HIIT program and the AE classification were assessed by a medical doctor.

### 2.3 Analysis

Tolerability of the HIIT program was assessed in the intention-to-treat (ITT) population defined as all included participants with a baseline observation. We used simple descriptive statistics (mean, standard deviation) for both the illustration of the trajectories and the tabulated results. Missing data for outcomes collected at baseline and after the 12 weeks were addressed using simple non-responder imputation: baseline observations carried forward. All data were analyzed using SAS version 9.4 (SAS Institute Inc., Cary, NC, USA).

## 3 Results

**Fig. 1:**
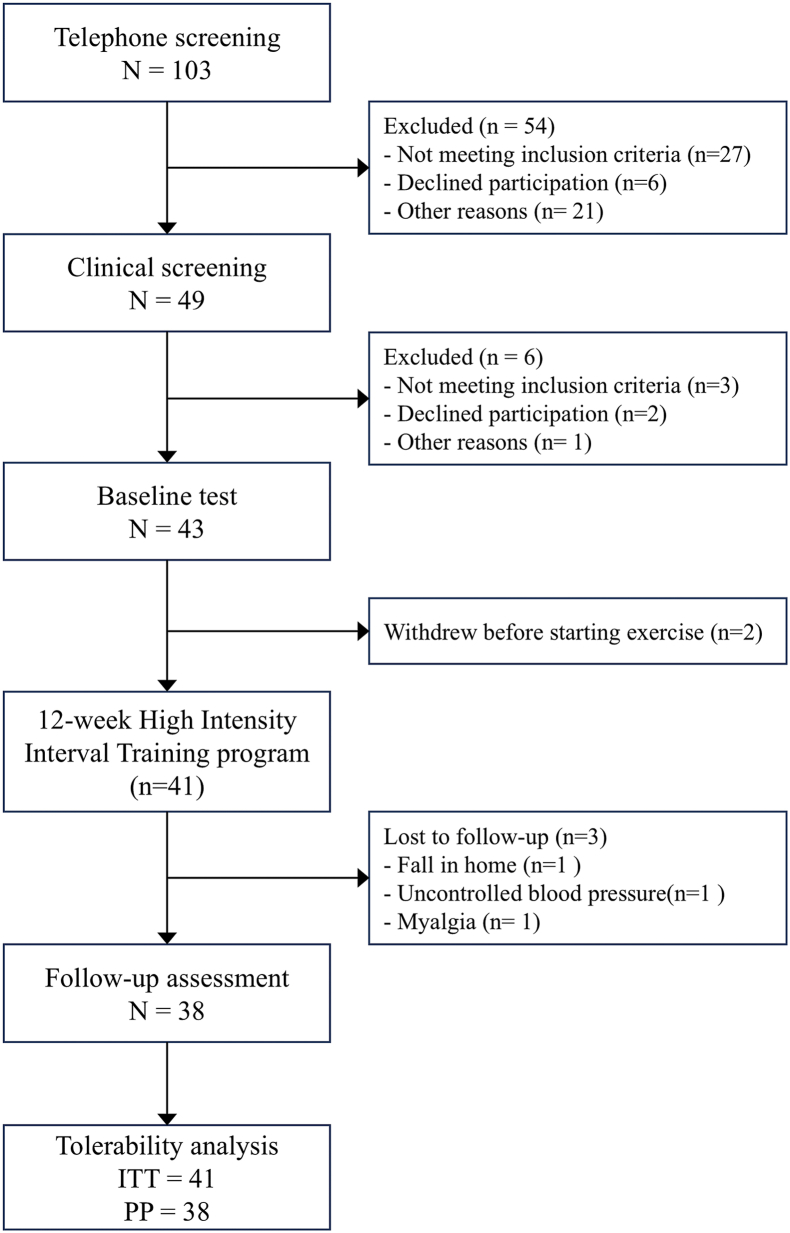
Participant flow The figure illustrates participant progression through each study phase, including telephone and clinical screening, baseline testing, completion of the 12-week HIIT exercise program, follow-up assessment, and inclusion in the tolerability analysis.

Participants were recruited from March 1st, 2023, to November 2nd, 2024. A total of 103 individuals were screened of which 43 were eligible and completed baseline assessments, 41 initiated the 12-week HIIT program, and 38 completed follow up assessments at week 12. Three participants discontinued participation for reasons not related to the HIIT program (Fig. 1). Table 2 summarizes participants' baseline demographics and clinical characteristics. The mean age was 72.4 years, with 66 % (n = 27) being female. The average BMI was 29.5 kg/m^2^), and 37 % (n = 15) were classified as obese (BMI ≥30 kg/m^2^). Participants had mild difficulty in Pain, Activities of Daily Living (ADL), and Symptoms; moderate difficulty in Knee-related Quality of Life; and moderate-to-severe difficulty in Function in Sports and Recreation, based on KOOS subscale scores.

**Table 2:** Baseline demographic and clinical characteristics of the participants in the intention to treat (ITT) population (n = 41).

Characteristics	Mean (SD)	
Age, years	72.4 (5.6)	
Sex, female, *n* (%)	27 (66 %)	
Height (cm)	167.2 (8.8)	
Body weight (kg)	82.8 (17.6)	
BMI kg/m^2^	29.5 (5.2)	
*Cardiovascular risk factors*		
HbaA1c (mmol/mol)	39.5 (4.6)	
HbaA1c > 44 mmol/mol, n (%)	6 (15 %)	
Blood pressure systolic (mm Hg)	141.4 (14.5)	
Blood pressure diastolic (mm Hg)	80.8 (10.0)	
Hypertension^a^, n (%)	14 (34 %)	
Cholesterol LDL mmol/L	2.8 (0.9)	
Cholesterol LDL >3.0 mmol/L, n (%)	16 (39 %)	
Triglycerides mmol/L	1.2 (0.5)	
Triglycerides >1.7 mmol/, n (%)	5 (12 %)	
BMI ≥30 kg/m2, n (%)	15 (37 %)	
Cardiorespiratory fitness (VO_2_max), ml/min/kg	22.3 (6.0)	
*Analgesic use*		
Paracetamol or NSAIDs user, n (%)	24 (59 %)	
*KOOS (0–100 scale)*^b^		*KOOS severity categories*^c^
Pain	61.7 (15.8)	Moderate difficulty
Function in daily life	66.6 (15.8)	Mild difficulty
Knee related quality of life	40.4 (15.4)	Mild difficulty
Function in sports and recreation	30.7 (22.2)	Moderate-severe difficulty
Symptoms	64.0 (16.3)	Moderate difficulty

Abbreviations; BP, Blood pressure; BMI, Body Mass Index; KOOS, The Knee injury and Osteoarthritis Score.

Data are presented mean ± standard deviation unless otherwise indicated.

This table presents participant demographics and baseline clinical measures, providing an overview of the study population prior to the intervention.

a Hypertension is defined as elevated blood pressure): Systolic *≥* 135 and/or diastolic *≥* 85 mm Hg.

b Scores on KOOS subscales range from 0 (worst) to 100 (best).

c KOOS severity categories based on Roos EM (2024): 0 = extreme difficulty, 25 = severe, 50 = moderate, 75 = mild, 100 = no difficulty. Severity classification is based on the mean subscale score.

**Fig. 2:**
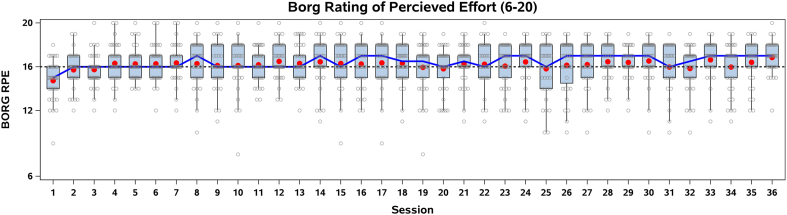
Average perceived exertion during interval training using Borg Rated Perceived Exertion (RPE) (6–20). The box plots depict the distribution of perceived exertion scores, with interquartile ranges (boxes), 90 % whiskers, and individual participant data (open circles). Means are illustrated as red dots and medians as the blue line. The target intensity of 16 Borg RPE is shown as a black dashed reference line.

Fig. 2 illustrates the Borg (RPE) across the 36 interval exercise sessions. Exercise intensity, as measured by the Borg Scale (6–20), averaged 16.2 (SD1.2) over the 12 weeks. Fig. 2 also illustrates the familiarization of the HIIT program in the first two weeks, (i.e., Borg RPE >16 during the intervals). The mean (red dots) and median Borg RPE (blue line) indicate a consistent perceived exertion level throughout the 12 weeks, generally fluctuating around 15–17 on the Borg scale. The majority of exercise sessions (69 %) were performed on an exercise bike followed by row ergometer, cross-trainer and treadmill (Supplement 2).

### 3.1 Tolerability of the HIIT program

The tolerability data are summarized in Table 3 and was evaluated using the Decision Guiding framework (DGF; Table 1) and discussion among the investigators.

**Table 3:** Tolerability-data in the intention-to- treat (ITT) population (n = 41).

		Consensus Evaluation
**Attendance**		
Average attendance to exercise sessions (out of 36), mean (SD)	31.9 (6.9)	
Attendance to education session (out of 1), n (%)	41 (100 %)	
**Knee pain flares**		
Knee pain flares the first 6 weeks, n (N, %)^a^	3 (3,7 %)	
Knee pain flares the last 6 weeks, n (N, %)^a^	5 (4, 10 %)	
Difference in knee pain flares from the first to the last 6 weeks	2	
**Change in knee pain at exercise session start (0–10 NRS) from week 1 to week 12**		
Number of participants with ≥1 point knee pain reduction, n (%)	19 (46 %)	
Number of participants with ≥1 point knee pain increase, n (%)	5 (12 %)	
Average change in weekly knee pain	−0.9 (1.4)	

The table presents attendance rates, frequency of knee pain flares, and changes in knee pain over the 12-week intervention period, indicating overall program tolerability.

a Numbers of knee pain flares resulting in non-compliance with/modification of the HIIT exercise program (n) in number of participants (N, %).

All participants (100 %) attended the educational session, and the average attendance to the HIIT program was 31.9 sessions (SD 6.9) out of 36. By consequence the attendance domain of the DGF was considered to support tolerability, which was agreed upon by all investigators.

There were eight knee pain flares resulting in non-compliance or modification of the HIIT program reported by six participants (15 %). Three flares occurred in weeks 1–6 in three participants, and these were all transient and resolved spontaneously over one to two days. During weeks 7–12, five knee pain flares occurred in four participants (one participant experienced a flare in both halves of the intervention). The flares were transient with spontaneous recovery over on to two days, and two were considered related to pre-existing Baker's cysts. One of the knee pain flares led to discontinuation in week 10–12 in the HIIT program During pain flares, exercise intensity was modified from the target to between 12 and 14 on Borg RPE. According to the DGF, the data suggested the HIIT program to be “not tolerable” as the incidence of flares did not decrease over the 12-week period. However, the investigators reached a consensus to upgrade it to “tolerable for most people” (“tolerable” or “partly tolerable”) due to the low incidence of flares occurring in only a few participants over a large number of exercise sessions, the mainly transient and benign nature of the flares, and the minor impact on the overall intensity of the HIIT program. Furthermore, sporadic and transient increases in knee OA pain are frequently occurring as part of the natural course of the disease. The exercise intervention was delivered as planned with only small deviations such as duration of each session.

**Fig. 3:**
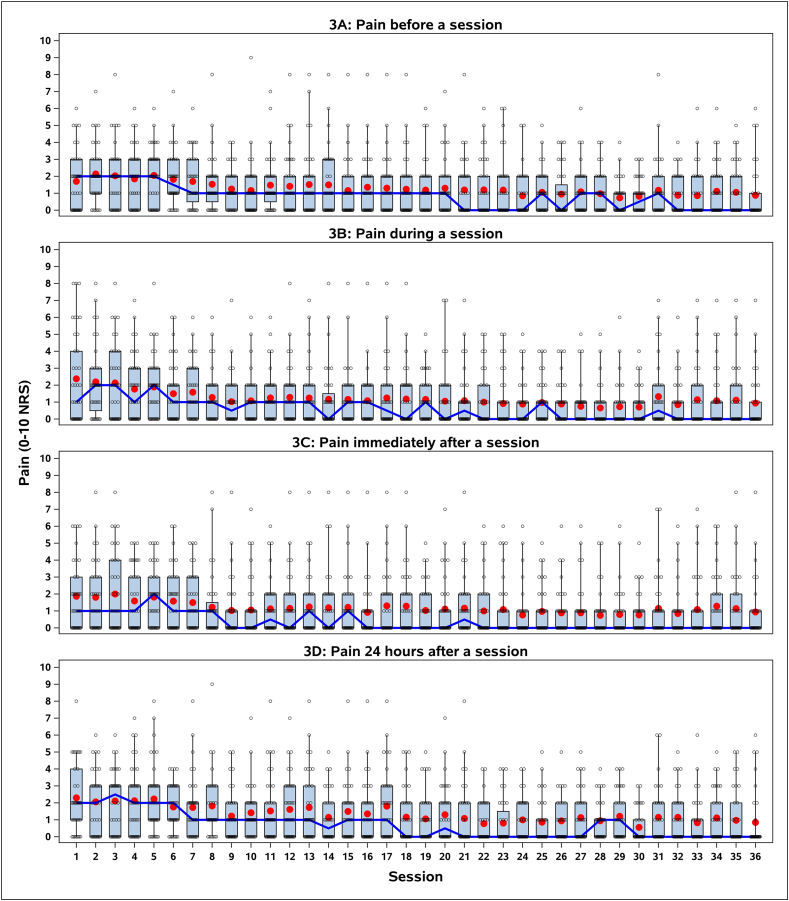
Trajectories of various knee pain scorings during the 12 weeks of High Intensity Interval Training (HIIT) exercise. A: Knee pain when starting exercise sessions. B: Knee pain during exercise sessions. C: Knee pain after exercise sessions. D: Knee pain 24 h after exercise sessions. The plots depict the distribution of knee pain scores, with interquartile ranges (boxes), 90 % whiskers, and individual participant data (open circles). Means are illustrated as red dots and medians as the blue lines.

Fig. 3 presents pain levels before, during, immediately after, and 24 h after an exercise session reported by participants across the 36 exercise sessions using the Numeric Rating Scale (NRS, 0–10). The figure indicates that knee pain levels fluctuate slightly with a trend towards lower scores over time. For the change in knee pain domain of the tolerability assessment, 19 participants (46 %) reported at least a one-point reduction (on a 0–10 scale) at exercise session start from week one to week 12 and five participants (12 %) reported a one-point pain worsening when starting an exercise session. In the last week (week 12) 19 participants (46 %) reported no pain at exercise session start. The median pain rating at exercise session-start in the first week was two, and zero in the last week (Fig. 3A). The average change in knee pain at exercise session start from week 1–12 was −0.9 (SD 1.4). According to the DGF the data suggested “partly tolerable”, however, the investigators reached consensus to upgrade to “tolerable” based on the low pain scores in week 1, the overall reduction, and the number of participants reporting no pain at week 12.

Based on the combined domain-level assessments and the investigator consensus discussions, the HIIT intervention was classified as “tolerable for most people with knee OA.

### 3.2 Clinical outcomes

The study observed changes across several clinical outcome measures following the 12-week HIIT program (Table 4). There was a reduction in body weight (−0.9 kg, 95%CI: 1.7 to −0.2) and BMI (−0.3 kg/m^2^, 95 % CI: 0.6 to −0.04). Systolic blood pressure decreased by 9.1 mmHg (95 % CI: 14.9 to −3.2), and diastolic blood pressure decreased by 3.6 mmHg (95%CI: 6.8 to −0.6). Cardiorespiratory fitness (VO2max), increased by 1.8 ml/min/kg (95%CI: 1.1 to 2.6) and improvements in all KOOS subscales; Pain 10.3 (95%CI: 5.2 to 15.4), Function in daily life 10.04 (95%CI: 5.6 to 14.7), Knee related quality of life 8.8 (95%CI: 3.9 to 13.8), Function in sports and recreation 9.6 (95%CI: 3.3 to 15.9), and Symptoms 9.1 (95%CI: 3.8 to 14.5). Furthermore, of the 24 participants who used analgesics at baseline 17 (71 %) discontinued the use of paracetamol or NSAIDs.

**Table 4:** Change from baseline to week 12 in clinical outcomes.

	Mean (95 % CI)
Body weight (kg)	−0.9 (−1.7 to −0.2)
BMI (kg/m^2^)	−0.3 (0.6 to 0–04)
*Cardiovascular risk factors*	
HbaA1c (mmol/mol)	0.4 (0.1–0.9)
Blood pressure systolic (mm Hg)	−9.1 (−14.9 to -3-2)
Blood pressure diastolic (mm Hg)	−3.6 (−6.8 to −0.6)
Cholesterol LDL mmol/L	0.0 (−0.1 to 0.1)
Triglycerides mmol/L	0.0 (−0.1 to 0.1)
Cardiorespiratory fitness (VO2max), ml/min/kg	1.8 (1.1–2.6)
*Analgesic use*	
Paracetamol or NSAIDs discontinued, n (%-points)	17 (−71 %-points)
*KOOS (0–100 scale)*^a^	
Pain	10.3 (5.2–15.4)
Function in daily life	10.0 (5.6–14.7)
Knee related quality of life	8.8 (3.9–13.8)
Function in sports and recreation	9.6 (0.3.3 to 15.9)
Symptoms	9.1 (3.8–14.5)

Abbreviations: SD, Standard Deviation; BP, Blood pressure; BMI, Body Mass Index; KOOS, The Knee injury and Osteoarthritis Score.

Data are presented as mean change from baseline (95 % Confidence Interval) unless otherwise stated.

The table presents changes in body composition, cardiovascular risk factors, cardiorespiratory fitness, analgesic use, and KOOS subscale scores, summarizing the clinical effects of the intervention.

a Scores on KOOS subscales range from 0 (worst) to 100 (best).

### 3.3 Safety during the 12-week HIIT intervention

During the 12-week HIIT program, the total exposure time was 471 participant weeks (Table 5). Adverse events (AEs) were reported in 17 participants (41.5 %), with a total of 19 events, corresponding to a rate of 0.04 events per patient week. AEs led to the discontinuation of the program in 3 participants (7.3 %) (Fig. 1). Most AEs were classified as moderate (13 events, 31.7 %), with fewer events classified as mild (3 events, 7.3 %) or severe events (1 event, 2.4 %). Most AEs (13 events) were not related to the HIIT program, while 6 events were classified as probably related. No serious adverse events (SAEs) were reported during the program (Table 5).

**Table 5:** Adverse events during the 12-week High Intensity Interval Training (HIIT) exercise program.

	N = 41
Exposure time – patient weeks	471
AE - no. of patients (%)	17 (41.5 %)
AE - no. of events (rate-events per patient week)	19 (0.04)
AEs leading to intervention discontinuation - no. of patients (%)	3 (7.3 %)
Max. Reported severity of AEs^a^	
Mild - no. of patients (%)	3 (7.3 %)
Moderate - no. of patients (%)	13 (31.7 %)
Severe - no. of patients (%)	1 (2.4 %)
AEs, relationship to trial treatment	
Not related **-** no. of events	13
Probably related **-** no. of events	6
AEs, classification,	
Cardiovascular and orthostatic condition – no of events	3
General conditions – no of events	5
Musculoskeletal and connective tissue disorders – no of events	7
Injuries– no of events	4
SAE, no of events (rate-events per patient week)	0 (0)
Deaths **-** no. of events	0 (0 %)

The table summarizes the number, severity, classification, and relatedness of adverse events, describing the overall safety profile of the intervention.

AE, adverse event; SAE, serious adverse event.

a The severity of an AE refers to the maximum intensity of the event. An event was considered severe (compared with mild or moderate) if it interfered substantially with the patients' usual activities.

## 4 Discussion

This prospective cohort study explored the tolerability of a 12-week HIIT program in individuals with knee OA and CVD risk factors. The main findings were that the program was well tolerated, with high attendance, few transient knee pain flares, and clinically relevant improvements in knee pain and patient-reported outcomes. During the 12 weeks, 46 % of participants reported at least a one-point pain reduction from the first to the last week when starting an exercise session. For the KOOS pain there was a reduction of approximately 10 points over the 12 weeks, which is similar to other types of exercise interventions [20,29,30].

Interpreting the KOOS scores using the recently published severity thresholds (Roos, 2024) provides important context for understanding our findings [31]. At baseline, participants reported mild difficulty on KOOS Pain, Symptoms, and ADL, but moderate difficulty in knee-related quality of life and moderate-to-severe difficulty in Sport/Rec, indicating that the population entered the trial with meaningful functional limitations despite relatively mild pain levels. Interpreting changes over time within this severity framework may help clarify the clinical relevance of the observed effects.

The feasibility of the HIIT program was supported by consistent exercise intensity, as indicated by the Borg RPE Scale, which were within the targeted range of moderate to high intensity throughout the 12 weeks. The high attendance rates and perceived exercise intensities suggest that participants were able to follow the prescribed HIIT program, reinforcing its feasibility in a clinical setting. However, individual variability in perceived exertion highlights the need for adaptive exercise prescriptions to optimize both engagement, adequate intensity, and tolerability [18].

The use of the DGF for the tolerability assessment provided valuable insight into the tolerability of HIIT. Attendance was rated as “tolerable” due to the high participation rate, reinforcing the feasibility of HIIT in this population and suggesting that the HIIT program did not interfere with the participants’ daily life. This aligns with findings from other studies reporting high adherence to supervised exercise interventions for knee OA, reinforcing the acceptability of structured exercise programs in this population [29,32,33].

The knee pain flare data initially indicated “not tolerable” as the incidence of knee pain flares leading to exercise modifications was stable over the period. However, the knee pain flares were few and isolated to six participants. Moreover, they were transient, and led to minor reductions in the exercise intensity and did not lead to discontinuation of the exercise program. Nevertheless, this highlights the need for personalized adjustments and careful monitoring in HIIT programs among persons with knee OA, especially if Baker's cysts are present. While exercise-induced symptom fluctuations are expected in OA, knee pain flares leading to exercise modifications are rarely reported in exercise studies, making it difficult to compare the incidence with other studies. This highlights the need for confirmatory studies—including those with placebo or attention control groups—to better contextualize and interpret the frequency and clinical relevance of such flares. One study reported transient joint pain increases during an 8-week neuromuscular exercise program, but these acute exercise-induced pain flares decreased over time [34]. While the incidence of knee pain flares in our study was low, it is notable that a previous study on neuromuscular training reported a higher proportion of participants requiring exercise modification (28 %) [30]. In this perspective, we reconsidered the knee pain flare criterion to be upgraded to “tolerable for most people with knee OA”. (18).

Knee pain was initially rated “partly tolerable” as 46 % of the participants had more than 1 point reduction in weekly average knee pain when starting an exercise session from the first to the last week. However, the pain levels at session start were low (week 1: 2.0, SD 1.6), and by week 12, almost 50 % of participants reported no pain. The low initial pain levels impede the possibility to assess tolerability based on pain improvement. However, only five participants experienced worsening of pain of more than one point throughout the 12 weeks, and these worsening's were minor. Also, we found a substantial number of participants that discontinued analgesics, and only one participant started analgesics use during the 12 weeks. This suggests that the pain improvements were not driven by analgesics use. With these perspectives in mind, we upgraded the pain criterion to “tolerable”.

The modest pain reduction observed in this study aligns with findings from another assessment of a HIIT intervention for knee OA [20]. That study found that pain levels remained stable or improved slightly, reinforcing the notion that HIIT does not typically exacerbate symptoms in the short term. Similarly, the START trial, which investigated high-intensity strength training in knee OA, found no significant additional pain relief compared to lower-intensity exercise, suggesting that pain reduction is not solely dictated by exercise intensity [29]. This is further supported by our KOOS findings, where improvements in pain were comparable to other exercise interventions, such as those in the DISCO and the START trials [29,33]. These findings suggest that HIIT may not only be tolerable but could also contribute to symptom relief, warranting further investigation. Furthermore, the substantial proportion of participants who discontinued analgesic use suggests that HIIT may contribute to pain management, potentially reducing reliance on medication. This has important clinical implications, as long-term analgesic use, particularly NSAIDs, is associated with cardiovascular risks in older adults [35].

The 12-week HIIT program resulted in improvements in health outcomes. Cardiorespiratory fitness increased, which aligns with previous findings [20] and a narrative review highlighting that HIIT can enhance aerobic capacity, muscle strength, and quality of life in knee OA patients, with few adverse events [36]. We observed significant reductions in blood pressure following the intervention, with mean decreases of 9.1 mmHg in systolic and 3.6 mmHg in diastolic measurements. These reductions are comparable to those reported in meta-analyses of HIIT training in non-osteoarthritis (OA) populations, demonstrating a reduction in systolic blood pressure ranging from approximately 3 to 7 mmHg and a diastolic reduction of about 2–5 mmHg. Such decreases in blood pressure are clinically meaningful, as evidence indicates that a 10-mmHg reduction in systolic blood pressure is associated with a 20 % lower risk of major cardiovascular events, including coronary heart disease and stroke [37,38]. Therefore, our findings suggest that HIIT may contribute to reducing CVD risk in patients with knee OA.

In terms of body weight and metabolic markers, our study observed minimal changes in weight loss, HbA1c, LDL cholesterol, and triglycerides, which highlight the potential need for combined lifestyle interventions to optimize metabolic health improvements in patients with knee OA [39].

The safety of the 12-week HIIT program was generally acceptable, with a total of 19 adverse events (AEs) reported in 17 participants (41.5 %), corresponding to 0.04 events per patient-week. Notably, no serious adverse events (SAEs) or deaths were recorded, indicating that HIIT can be a viable exercise option for individuals with knee OA and increased CVD risk. Compared to previous HIIT studies in OA populations, the observed AE rate is consistent with previous reports of no serious adverse events and a low dropout rate, suggesting that HIIT can be tolerated with proper modifications [20]. Similarly, the START trial investigating high-intensity strength training in patients with knee OA found a higher rate of musculoskeletal complaints compared to a low-intensity program, emphasizing the importance of individualized exercise prescription [29]. While musculoskeletal AE is a known risk in OA exercise interventions, structured modifications, such as adjusting exercise volume, intensity, or recovery periods, may help reduce musculoskeletal AEs while maintaining the benefits of high-intensity training [30].

This study also has limitations. First, the lack of a control group limits the ability to attribute observed improvements solely to the HIIT program. Second, there is a potential for selection bias, as participants who chose to enroll may have been more motivated or more physically capable than the general knee OA population. Third, we did not include an objective measure of exercise intensity; however, the observed improvement in cardiorespiratory fitness and blood pressure suggests that the self-reported intensity was sufficient to elicit a physiological training effect. Fourth, we did not collect information on concurrent treatments or lifestyle changes outside the intervention, which represents a potential source of confounding. Additionally, although predefined guided tolerability interpretation, the thresholds for knee pain reduction and flare incidence may not fully capture individual tolerability.

The present study has several strengths; A key strength of this study is the use of the decision guidance framework, which provided a structured approach to evaluating the tolerability of a HIIT program in people with knee OA and CVD risk factors. Additionally, the study employed a pragmatic design, incorporating self-selected exercise modalities to enhance real-world applicability. Furthermore, the supervision and coaching during the 12-week HIIT program ensured, that participants performed at intended exercise intensity and the safety of the HIIT program.

Our findings indicate that HIIT may be a feasible and tolerable exercise option for individuals with knee OA and cardiovascular risk factors. Future randomized controlled trials are warranted to confirm these results, include objective intensity measures, and explore long-term effects on cardiovascular and joint health.

## 5 Conclusion

This study suggests that the HIIT program is tolerable for most individuals with knee OA and CVD risk factors including those with mild-to-moderate functional limitations. While some participants experienced symptom exacerbations requiring exercise modifications, these were generally few and transient. Careful monitoring of knee pain flares and individualized adjustments to exercise remain important to support tolerability across a broader patient population. In addition, the HIIT program was associated with improvements in cardiorespiratory fitness, blood pressure, knee function, and patient reported outcomes.

## Author contributions

M. Henriksen, T. Schjødt Jørgensen, T. Bandholm, M. Ried-Larsen, and C. Bartholdy conceived the study and developed the design of the study. M. Pedersen analyzed the data and drafted the first version of the manuscript. All authors critically reviewed and revised the manuscript and approved the final manuscript and submission of the article. M. Henriksen and M. Pedersen had full access to all data in the study.

## Funding

The study was supported by Sundheds donationer under the Danish health insurance company Sygeforsikring “danmark” (grant no. 2021-0025). The Parker Institute, Bispebjerg and Frederiksberg Hospital, is supported by a core grant from The Oak Foundation (grant no. OFIL-24-074), of which a not specified amount may be used to cover expenses in this study. The funders had no role in the study due to study design and conduct, analyses, and interpretation of the data; development, review, and/or approval of the manuscript, submission, and choice of publication journal.

## Conflict of interest statement

All authors have completed the ICMJE Disclosure Form, submitted with the manuscript and available from corresponding author.
