# Supplementary material for: Tolerability of high intensity interval training (HIIT) in individuals living with knee OA and at risk for cardiovascular disease: A prospective cohort study (THIPO)
Source: Osteoarthr Cartil Open. 2026 Jan 12;8(1):100712. doi: 10.1016/j.ocarto.2025.100712 (PMC12861021; doi:10.1016/j.ocarto.2025.100712)
Supplement: Multimedia component 1 [file mmc1.docx]

| **Section/Topic** | **Item #** | **Checklist item** | **Location (Primary paper, page, table, appendix** | |
| --- | --- | --- | --- | --- |
| WHAT: materials | 1 | Detailed description of the type of exercise equipment (e.g. weights, exercise equipment such as machines, treadmill, bicycle ergometer etc) | The HIIT exercise program was performed on treadmills, cross-trainers, row- and cycle ergometers, as the participants preferred. This approach facilitated individualization, accommodating differences in physical ability, joint discomfort, and exercise preferences. By providing multiple exercise options, the program aimed to enhance adherence by involving participants in the choice of modality. | Page 5 |
| WHO: provider | 2 | Detailed description of the qualifications, teaching/supervising expertise, and/or training undertaken by the exercise instructor | The HIIT exercise program was delivered and by an exercise trainer. | Page 5 |
| HOW: delivery | 3 | Describe whether exercises are performed individually or in a group | The exercises were performed in groups of 1-5 persons. | Page 5 |
|  | 4 | Describe whether exercises are supervised or unsupervised and how they are delivered | The exercises were supervised by exercise physiologists /trainers. | Page 5 |
|  | 5 | Detailed description of how adherence to exercise is measured and reported | Attendance to each session were recorded as participants meeting at the gym. The exercise trainers recorded whether the HIIT exercise program was completed (yes/no), minutes of exercise, type of equipment, and average self-perceived exertion using the 6-20 Borg RPE. | Page 5 |
|  | 6 | Detailed description of motivation strategies | The HIIT exercise program was performed on treadmills, cross-trainers, row- and cycle ergometers, as the participants preferred. This approach facilitated individualization, accommodating differences in physical ability, joint discomfort, and exercise preferences. By providing multiple exercise options, the program aimed to enhance adherence by involving participants in the choice of modality. | Page 5 |
|  | 7a | Detailed description of the decision rule(s) for determining exercise progression | If the participant was able to talk (Borg RPE <16), the was participant was encouraged to increase either speed or resistance. | Page 5 |
|  | 7b | Detailed description of how the exercise program was progressed | If the participant was able to talk (Borg RPE <16), the participant was encouraged to increase either speed or resistance. | Page 5 |
|  | 8 | Detailed description of each exercise to enable replication (e.g. photographs, illustrations , video etc) | The HIIT exercise program was performed on treadmills, cross-trainers, row- and cycle ergometers, as the participants preferred | Page 5 |
|  | 9 | Detailed description of any home program component (e.g. other exercises, stretching etc) | There was not provided any home-program component. | Page 5 |
|  | 10 | Describe whether there are any non-exercise components (e.g. education, cognitive behavioural therapy, massage etc) | The HIIT program consisted of a 12-week education and supervised HIIT program. The education session was delivered by a physiotherapists or/and exercise physiologists at The Parker Institute at Bispebjerg Frederiksberg Hospital, in one 1-hour session, in groups of 1-5 persons, and focused on physiological effects of aerobic exercise, recommendations for diet during an exercise program, potential experience of delayed onset muscle soreness, management of potential knee | Page 5 |
|  | 11 | Describe the type and number of adverse events that occurred during exercise | During the 12-week HIIT-intervention, the total exposure time was 471 participant weeks (Adverse events (AEs) were reported in 17 participants (41.5%), with a total of 19 events, corresponding to a rate of 0.04 events per patient week. AEs led to the discontinuation of the intervention in 3 participants (7.3%) (Figure 1). The majority of AEs were classified as moderate (13 events, 31.7%), with fewer events 3 events, 7.3%) or severe (1 event, 2.4%). Most AEs (13 events) were not related to the HIIT program, while 6 events were classified as probably related. No serious adverse events (SAEs) or deaths were reported during the intervention (Table 5). | Table 5, page 17 |
| WHERE: location | 12 | Describe the setting in which the exercises are performed | The exercise program was performed at The Parker Institute at Bispebjerg Frederiksberg Hospital. | Page 5 |
| WHEN, HOW MUCH: dosage | 13 | Detailed description of the exercise intervention including, but not limited to, number of exercise repetitions/sets/sessions, session duration, intervention/program duration etc | The HIIT exercise program was delivered by an exercise trainer in 45-minute supervised sessions 3 times/week in groups of 6-12 participants for 12 weeks. The standardized HIIT exercise program comprised a 10-minute warm-up, followed by eight intervals; 2-minute intervals of high intensity training (aiming a heart rate of at least 80% of HRmax – corresponding to Borg RPE of at least 16 as an average of the high intensity intervals) followed by 2 minutes of moderate training intensity (aiming a heart rate of at least 60% of HRmax corresponding to a Borg RPE of 11), and five minutes of cool down. In the first two weeks of the HIIT intervention, a familiarization to the HIIT exercise program occurred (i.e., Borg RPE <16 during the intervals was expected and allowed in this period) to ensure good exercise quality and motivation in the remaining part of the exercise program.  Exercise program | Page 5 |
| TAILORING: what, how | 14a | Describe whether the exercises are generic (one size fits all) or tailored whether tailored to the individual | All participants followed the same standardized HIIT program; however, individual tailoring was applied based on knee symptoms and tolerability. While all participants were encouraged to perform the sessions as protocolized, adjustments were made and recorded in response to knee pain, flares or other physical limitations. | Page 5 |
|  | 14b | Detailed description of how exercises are tailored to the individual | If a participant reported knee pain of ≥5 before or during a session, the exercise was moderated (e.g., reduced resistance) to maintain the prescribed exertion level while lowering joint load. In case of a pain flare, modifications were applied in subsequent sessions. If the pain level was ≥5 at baseline but considered usual for the participant, the session proceeded as planned unless symptoms worsened, prompting adjustments. Modifications to the HIIT program included reducing resistance, adjusting speed, or substituting exercise equipment to maintain the target exertion level while minimizing joint stress. This approach ensured the intervention remained both standardized and responsive to individual needs. | Page 6 |
|  | 15 | Describe the decision rule for determining the starting level at which people commence an exercise program (such as beginner, intermediate, advanced etc) | In the first two weeks of the HIIT intervention, a familiarization to the HIIT exercise program occurred (i.e., Borg RPE <16 during the intervals was expected and allowed in this period) to ensure good exercise quality and motivation in the remaining part of the exercise program. | Page 5 |
| HOW WELL: planned, actual | 16a | Describe how adherence or fidelity to the exercise intervention is assessed/measured | Adherence and fidelity to the HIIT intervention were assessed by study personnel who documented whether each exercise session was completed as per protocol (yes/no), based on predefined criteria outlined in Table 1 (main paper). This included recording the type of exercise equipment used, session duration, average Borg Rating of Perceived Exertion (RPE), and self-reported knee pain before, during, after, and 24 hours following each session using a 0-10 NRS. This comprehensive tracking ensured accurate monitoring of both adherence and deviations from the prescribed HIIT protocol. | Page 5 |
|  | 16b | Describe the extent to which the intervention was delivered as planned | The exercise intervention was delivered as planned with only small deviations such as duration of each session. | Page 13 |
