# Supplementary material for: Tolerability of high intensity interval training (HIIT) in individuals living with knee OA and at risk for cardiovascular disease: A prospective cohort study (THIPO)
Source: Osteoarthr Cartil Open. 2026 Jan 12;8(1):100712. doi: 10.1016/j.ocarto.2025.100712 (PMC12861021; doi:10.1016/j.ocarto.2025.100712)

**Tolerability of High Intensity Interval Training (HIIT) in individuals living with knee OA and at risk for cardiovascular disease: A prospective cohort study (THIPO)**

Mathilde Espe Pedersen, Thomas Bandholm, Mathias Ried-Larsen, Cecilie Bartholdy, Tanja Schjødt Jørgensen, Asbjørn Seenithamby Poulsen, Kasper Stagberg Madsen, Marius Henriksen

**Supplement 2**


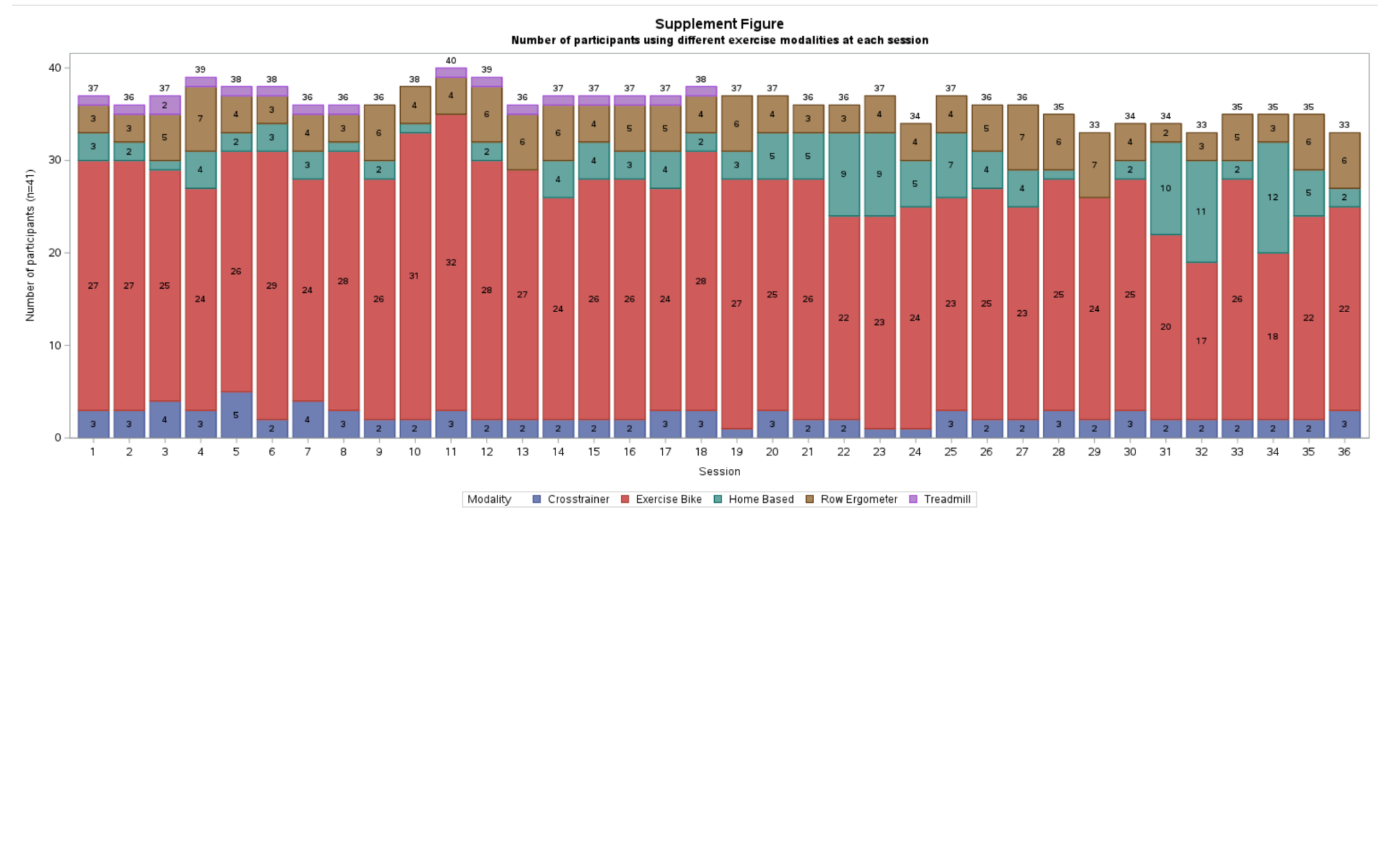

Supplement: Multimedia component 2 [file mmc2.docx]
